# Supplementary figures and images for: Phenotypic and Genotypic Diversity of Roots Response to Salt in Durum Wheat Seedlings
Source: Plants (Basel). 2023 Jan 16;12(2):412. doi: 10.3390/plants12020412 (PMC9865824; doi:10.3390/plants12020412)

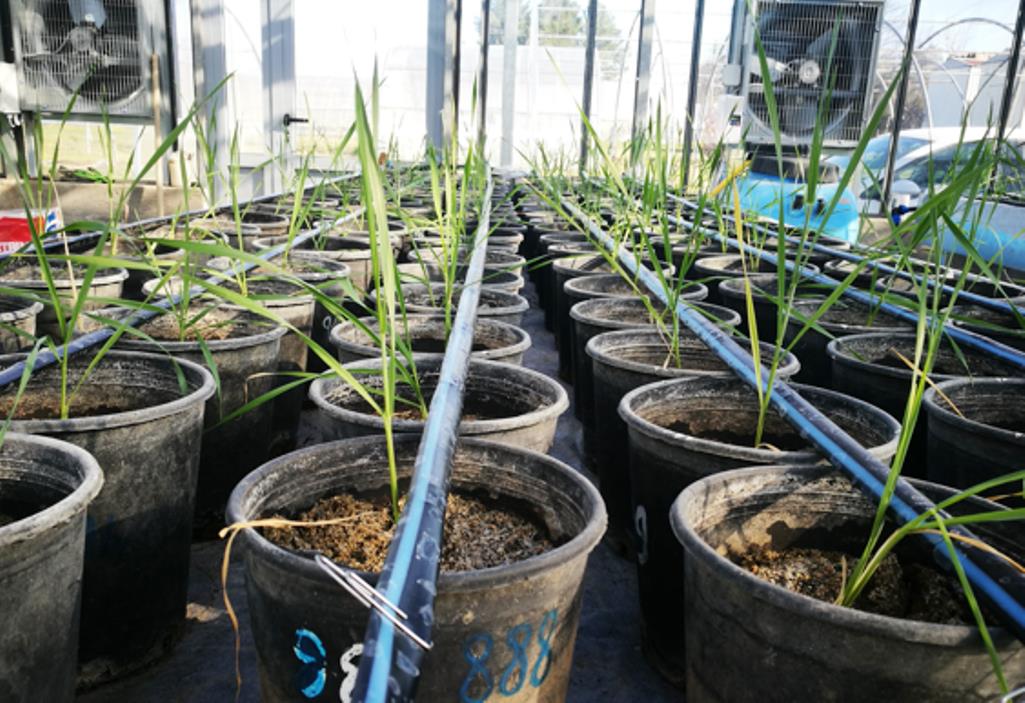

Supplement: Supplementary file 1 [file plants-12-00412-s001.zip › Figure S1.jpg]
